# Prognostic value of admission heart rate in patients with ST-segment elevation myocardial infarction: Role of Type 2 diabetes mellitus

**DOI:** 10.1186/1471-2261-12-104

**Published:** 2012-11-15

**Authors:** Zhang Han, Yang Yan-min, Zhu Jun, Liu Li-sheng, Tan Hui-qiong, Liu Yao

**Affiliations:** 1 Emergency and Intensive Care Center, Fuwai Hospital, Chinese Academy of Medical Sciences and Peking Union Medical College, Beijing, China

**Keywords:** Heart rate, ST-segment elevation myocardial infarction, Type 2 Diabetes Mellitus, Prognosis

## Abstract

**Background:**

It’s unknown whether the prognostic value of admission heart rate (HR) was different in patients with ST-segment elevation myocardial infarction (STEMI) with or without concomitant type 2 diabetes mellitus (T2DM).

**Methods:**

Consecutive STEMI patients who presented within 12 hours of symptom onset were recruited from 274 hospitals in China. Participants were stratified into quartiles by admission HR. Baseline characteristics, current therapeutic recommenda- tions, laboratory biochemical tests, 30-day all-cause mortality and Cardiovascular Events (CVE, including all-cause death, reinfarction and stroke) were compared across admission HR quartiles.

**Results:**

We evaluated 7294 STEMI patients, of these 820 (11.2%) had known T2DM. The admission HR quartile stratification was significantly associated with all-cause mortality and CVE regardless of T2DM status (P < 0.001 both for survival and CVE). After adjusted other risk factors, in patients without T2DM, comparing with HR <66 b.p.m., the increase of HR level was associated with worse prognosis (P < 0.05). In patients with T2DM, the hazard ratios for 30-day CVE were 1.75 (95%CI), 1.92 (95%CI), 3.00 (95%CI) in the HR of 66–76 b.p.m., 77–88 b.p.m., and >88 b.p.m., respectively. Results were similar for 30-day all-cause mortality, but the hazard ratios in Q2 (P = 0.139 and P =0.086 for survival and CVE, respectively) and Q3 groups were non-significant (P = 0.072 and P =0.033 for survival and CVE, respectively). There was a significant interaction effect of HR and T2DM on 30-day CVE mortality (P = 0.035), which was not found on all-cause mortality (P = 0.126).

**Conclusion:**

Admission heart rate was an important risk factor of 30-day all-cause mortality and CVE in patients with STEMI with or without T2DM. However, the predictive effect was modified by T2DM.

## Introduction

Several epidemiological and clinical studies have reported the association between heart rate (HR) and prognosis in general population and in patients with hypertension, stable coronary artery disease (CAD), heart failure (HF) [1-4], or acute coronary syndromes (ACS) [5,6].

In addition, the relationship between type 2 diabetes mellitus (T2DM) and prognosis has been well demonstrated in patients with acute myocardial infarction (AMI) [7,8]. Cardiac autonomic neuropathy (CAN) and higher HR may correlated with worse prognosis in diabetic patients after AMI [9,10]. However, in patients with ST-segment elevation myocardial infarction (STEMI), it is unclear whether the effect of HR on prognosis will be different between patients with and without T2DM.

The purpose of our report from a large study is to evaluate the association between admission HR and 30-day all-cause mortality and cardiovascular events (CVE) in STEMI patients with or without T2DM.

## Methods

### Patients

From July 2001 to July 2004, 7510 consecutive patients with a clinical diagnosis of STEMI were admitted to 247 hospitals throughout China within 12 h of the onset of symptom. Data were collected and recorded in a research database. AMI was diagnosed on the basis of the European Society of Cardiology and American College of Cardiology criteria [11]. STEMI was defined as AMI with ST-segment elevation in two contiguous leads or new left-bundle branch block on an ECG obtained at the time of admission. Only patients with sinus rhythm were included in analysis. Patients who were hemodynamically unstable and hospitalized with atrial fibrillation, second or third degree heart block, ventricular tachycardia, ventricular fibrillation and cardiac arrest were excluded. According to the exclusion criteria, 99 patients were excluded. 27 patients were excluded from analysis because of incomplete information.

### Data collection

At baseline, demographic characteristics and details of concomitant cardiovascular risk factors were recorded. Furthermore, a medical history was taken and fasting plasma glucose was assessed. HR was measured by a 12-lead ECG at admission. Qualifying patients received thrombolytic therapy or underwent percutanous coronary intervention (PCI) according to current therapeutic recommendations [12]. In-hospital and 30-day adverse events, including death, cardiogenic shock, HF, life-threatening arrhythmia and re-infarction were also recorded in detail.

The presence of known T2DM was recognized if the diagnosis was established according to the WHO definition [13] prior to enrollment, reported in the medical records, declared directly by the patient, or revealed by using the glucose-lowering medications, for example, metformin.

The follow-up duration was 30 days. Trained study personnel recorded all outcomes by reviewing the medical record, telephone or email contact. All-cause mortality and Cardiovascular Events (CVE, including all-cause death, reinfarction and stroke) in 30 days, were adjudicated by a central committee of clinicians.

The study was approved by the institutional Ethics committees at all participating hospitals. Informed consent was obtained from each patient.

### Study outcomes and definition

The primary outcomes were death from cardiac or non-cardiac cause in 30 days. CVE comprised of all-cause mortality, reinfarction, or stroke. Definitions of outcomes were as follows:

All-cause mortality included cardiovascular death (defined as any death with a cardiovascular cause including those deaths following cardiovascular procedures or surgery or deaths due to unknown cardiovascular cause) and non-cardiovascular death (defined as deaths due to a clearly documented non-cardiovascular cause).

Reinfarction was defined as the presence of at least two of the following criteria:

1. New onset of characteristic ischemic chest pain/symptoms occurring at rest or with minimal exercise;

2. Elevation of enzyme levels or markers (CK, CK-MB, other cardiac enzymes or Troponin I or T) to at least twice the upper limit of the normal reference range or if enzymes were already elevated, greater than 50% of the lowest recovery enzyme level from the index infarction;

3. New ECG changes compatible with ischemia.

Stroke was defined as the presence of a new focal neurologic deficit consistent with a vascular origin with signs or symptoms lasting > 24 hours. Confirmation by CT scan or MRI is was obtained when available.

### Statistical analysis

Continuous variables are expressed as mean ± standard deviation, and categorical variables as counts and percentages. Admission HR was firstly analyzed as a continuous variable, and then as a categorical variable (Q1:<66; Q2:66–76; Q3:77–88; Q4:>88b.p.m., beats per minute). Differences in baseline characteristics across admission HR quartiles were evaluated using the one-way analysis of variance (ANOVA) or Chi-square tests, according to T2DM status. The Kaplan–Meier curves were computed for cardiovascular mortality and CVE. The log- rank test was used to test the differences in the unadjusted survival curves. Hazard ratios (HRs) were estimated by the Cox proportional hazard regression model in patients with or without T2DM and the models were corrected for age, sex, systolic blood pressure(SBP), previous hypertension, myocardial infarction, heart failure (CHF), or stroke, ST-segment elevation leads, killip class, and intervention measures and drugs. Potential interaction (effect modification) between HR and diabetes was evaluated by adding a multiplicative interaction term (HR × diabetes) to the Cox model.

A two-sided P-value of 0.05 was considered statistically significant; all analyses were performed with SPSS 17.0 for Windows (SPSS Inc., Chicago, IL, USA).

## Results

**Figure 1:**
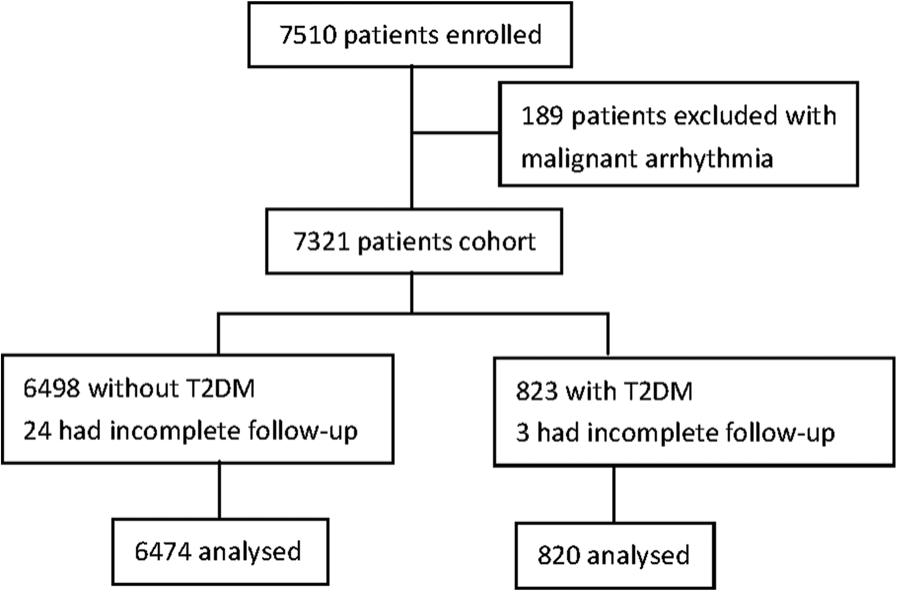
Flow of study patients.

Of the 7510 patients enrolled between July 2001 and July 2004 from 274 centers across China, data from 7294 patients (97.1%) were available for analysis, and 820 (11.2%) had known T2DM (Figure 1). Overall, median admission HR was 76 (65–88) b.p.m.; 76 (64–88) b.p.m. and 80 (68–94.5) b.p.m., respectively, in patients without and with T2DM.

### Baseline characteristics

Comparison of baseline demographic and clinical characteristics in hospital between patients with and without DM is detailed in Table 1. Patients with T2DM were older (64.7 ± 9.9 vs 62.3 ± 12.0, P < 0.001) and more of them were women (43.1% vs 27.1%, P < 0.001). More of the patients with T2DM were hypertensive (57.4% vs 38.5%, P < 0.001), had a previous stroke (16.5% vs 8.4%, P < 0.001), HF (6.3% vs 2.2%, P < 0.001) or myocardial infarction (12.2% vs 7.5%, P < 0.001) compared with patients without T2DM. T2DM was also associated with higher admission HR (82.2 ± 19.9 vs 77.2 ± 17.8, P < 0.001) and fasting serum glucose level (13.2 ± 5.5 vs 7.9 ± 3.6, P < 0.001) and more likely to be treated with insulin infusion (40.2% vs 11.5%, *P* < 0.05). Interestingly, participants with T2DM were more likely to receive PCI (16.9% vs 10.9%, P < 0.001), while fewer of them were treated with thrombolytic therapy (45.1% vs 53.3%, P < 0.001).

**Table 1:** Baseline characteristics and therapy of study participants according to diabetic state

	**Non T2DM(n** = **6474)**	**T2DM(n** = **820)**	**P**-**value**
Females, %	27.1	43.1	<0.001
Age	62.3 ± 12.0	64.7 ± 9.9	<0.001
Systolic blood pressure	126.5 ± 25.1	128.1 ± 26.0	0.039
Admission heart rate	77.2 ± 17.8	82.2 ± 19.9	<0.001
Anterior ST Elevation, %	53.4	52.6	0.633
Killip class II-IV, %	17.1	24.8	<0.001
Glucose Level, mmol/L	7.9 ± 3.6	13.2 ± 5.5	<0.001
**Previous medical history**			
Hypertension, %	38.5	57.4	<0.001
Myocardial Infarction, %	7.5	12.2	<0.001
Heart failure, %	2.2	6.3	<0.001
Stroke, %	8.4	16.5	<0.001
**Therapeutic measures**			
PCI, %	10.9	16.9	<0.001
Thrombolytic therapy	53.3	45.1	<0.001
Insulin	11.5	40.2	<0.001
β-blockers, %	62.1	63.3	0.506
ACEI, %	71.8	73.3	0.367
Lipid-lowering drugs, %	71.1	72.6	0.377

PCI, percutaneous coronary intervention; ACEI, Angiotensin-converting enzyme inhibitors.

Characteristics were reported in Table 2 by HR quartiles and by diabetic state. It’s similar that previous history of stroke and serum glucose level were not associated with admission HR (P > 0.05) in both diabetics and non-diabetics. Meanwhile, admission HR was associated with systolic blood pressure, frequency of anterior ST Elevation, Killip class II-IV, previous history of myocardial infarction or heart failure (*P* < 0.05). In addition, patients with a higher admission HR were were more likely to be treated with β-blockers, angiotensin-converting enzyme inhibitors (ACEI) and percutaneous coronary intervention (PCI) (*P* < 0.05) regardless of T2DM status.

**Table 2:** Baseline characteristics and therapy of non-diabetic patients based on quartiles of admission heart rate (Q1-Q4)

	**Patients without T2DM(n** = **6474)**				**P**-**value**	**Patients with T2DM(n** = **820)**				**P**-**value**
	**Q1(n** = **1766)**	**Q2(n** = **1696)**	**Q3(n** = **1518)**	**Q4(n** = **1494)**		**Q1(n** = **180)**	**Q2(n** = **175)**	**Q3(n** = **193)**	**Q4(n** = **272)**	
Females, %	25.3	25.5	26.8	31.3	<0.001	37.8	43.4	42.0	47.1	0.270
Age	62.3 ± 11.2	61.2 ± 11.9	61.7 ± 12.2	63.0 ± 12.9	<0.001	64.5 ± 9.9	64.0 ± 9.5	64.8 ± 10.2	65.3 ± 9.9	0.313
Systolic blood pressure	120.0 ± 24.0	128.0 ± 24.0	130.4 ± 23.3	128.5 ± 28.0	<0.001	120.2 ± 26.8	130.2 ± 22.0	130.6 ± 25.2	130.1 ± 27.4	<0.001
Anterior ST Elevation, %	34.4	51.6	62.7	68.5	<0.001	27.2	50.3	57.5	67.3	<0.001
Killip class ≥2, %	13.5	12.6	14.0	29.7	<0.001	15.6	13.7	20.2	41.2	<0.001
Glucose, mmol/L	7.8 ± 3.5	7.7 ± 3.2	7.8 ± 3.5	8.4 ± 4.2	0.097	13.0 ± 5.3	12.6 ± 5.0	12.8 ± 4.8	14.1 ± 6.2	0.054
**Previous medical history**										
Hypertension, %	36.4	36.9	38.7	42.6	0.001	55.6	53.1	53.9	64.0	0.062
Myocardial Infarction, %	7.3	6.5	6.8	9.6	0.005	6.7	9.7	11.9	17.7	0.003
Heart failure, %	1.0	1.2	2.0	5.0	<0.001	3.3	3.4	4.7	11.4	<0.001
Stroke, %	8.5	7.6	8.0	9.5	0.235	12.2	18.9	14.0	19.5	0.125
**Therapeutic measures**										
PCI, %	10.4	12.4	11.7	9.2	0.024	16.7	20.0	21.8	11.8	0.023
Thrombolytic therapy, %	57.6	54.7	54.2	45.6	<0.001	53.3	45.1	40.4	43.0	0.068
Insulin, %	11.4	9.5	11.8	13.8	0.002	42.8	37.1	37.9	42.3	0.548
β-blockers, %	45.8	67.3	69.5	68.0	<0.001	42.8	73.1	66.8	68.0	<0.001
ACEI, %	68.3	74.3	73.1	71.8	<0.001	63.3	73.1	75.7	78.3	0.004
Lipid-lowering drugs, %	69.7	73.6	71.6	69.3	0.023	71.7	76.0	73.1	70.6	0.644

PCI, Percutaneous coronary intervention; ACEI, Angiotensin-converting enzyme inhibitors; P-values are given for the comparison of between the different quartiles of heart rate.

In non-T2DM group, patients with higher admission HR were older, while more of them were women and hypertensive than those with lower admission HR (*P* < 0.05). They were more likely to use insulin, and less likely to be treated with thrombolytic therapy (P < 0.05). However, these differences across admission HR quartiles were not apparent in patients accompanied with T2DM.

### Primary and secondary outcomes

30-day outcomes were reported in Table 3 grouped by HR quartiles and by diabetic status. Patients with T2DM showed higher events rate for 30-day all-cause mortality (9.6% vs. 15.7%, *P* < 0.001) and CVE (11.2% vs. 18.1%, *P* < 0.001) compared with patients without T2DM. Events rate for 30-day all-cause mortality and CVE were higher with an increase of admission HR in patients independent of T2DM state (*P* < 0.001).

**Table 3:** 30-day events stratified by heart rate quartiles (Q1-Q4) in patients with and without T2DM

**Variable**	**Heart rate(b.p.m.)**					**P**-**value**
	**All**	**Q1**	**Q2**	**Q3**	**Q4**	
**Patients without T2DM**						
All-cause mortality (%)	9.6	6.9	7.0	8.0	17.3	<0.001
Cardiovascular events (%)	11.2	8.8	8.8	9.6	18.5	<0.001
**Patients with T2DM**						
All-cause mortality (%)	15.7	9.4	11.4	13.0	24.6	<0.001
Cardiovascular events (%)	18.1	10.6	12.6	16.0	27.9	<0.001

**Figure 2:**
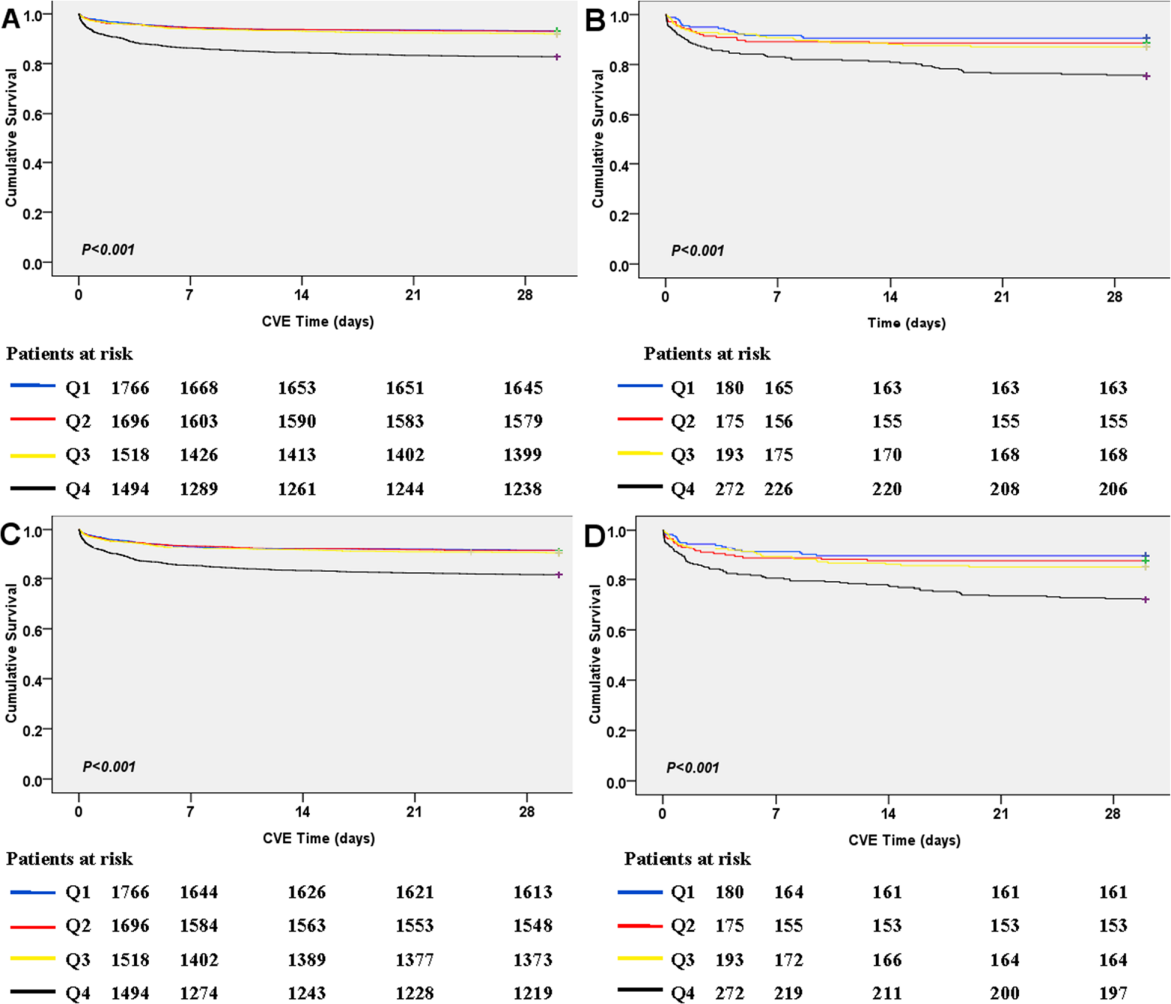
The Kaplan-Meier curves for survival (A and B) and survival free from CVE (C and D) by heart rate at admission in patients without (A and C) or with (B and D) T2DM (blue, Q1 ≤ 66 bpm; red, 66 < Q2 ≤ 76; yellow, 77 ≤ Q3 ≤ 88; black, Q4 > 88b.p.m.; P-value was calculated by log-rank test).

Kaplan-Meier curves for all-cause mortality and CVE were shown in Figure 2. The log-rank test reported that the unadjusted survival curves were not different among the lower three groups (*P* > 0.05, multiple comparison data not shown), while patients in the highest quartile of admission HR showed the highest rate of mortality and CVE (*P* < 0.001), regardless of T2DM.

Multivariable Cox regression analysis was performed to assess the prognostic value of admission HR after adjusting for age, sex, the medical history of hypertension, heart failure, myocardial infarction, stroke, Killip class, medications and procedures in-hospital (Insulin, β-blockers, ACEI, lipid-lowering drugs, and PCI). Analyses with HR as a continuous variable showed that admission HR was an independent risk factor of 30-day all-cause mortality (HR, 1.011; 95% CI, 1.008-1.015, *P* < *0*.*001*) and CVE (HR, 1.010; 95% CI, 1.006-1.013, *P* < *0*.*001*) in overall patients and in patients without T2DM (for all-cause mortality: HR, 1.013; 95% CI, 1.009-1.017, *P* < *0*.*001*; for CVE: HR, 1.010; 95% CI, 1.006-1.014, *P* < *0*.*001*). The similar predictive effect were found for 30-day CVE (P < 0.05).

Table 4 shows the details of association between quartiles of admission HR and prognosis. In patients without T2DM, comparing with HR <66 b.p.m., the HR of 66–76 b.p.m., 77–88 b.p.m., or >88 b.p.m. demonstrate Hazard Ratios of 1.44 (95% CI 1.13-1.84, P = 0.003), 1.41 (95% CI 1.10-1.80, P = 0.007), and 2.30 (95% CI 1.85-2.87, P < 0.001) for 30-day all-cause mortality; while hazard ratios for 30-day CVE were 1.39 (95% CI, 1.10-1.75, P = 0.005), 1.42 (95% CI 1.22-1.78, P = 0.003) and 2.30 (95% CI 1.87-2.83, P < 0.001). And in patients with T2DM, the hazard ratios for 30-day CVE were 1.75(95%CI), 1.92(95%CI), 3.00(95%CI) in the HR of 66–76 b.p.m., 77–88 b.p.m., and >88 b.p.m., respectively. Similar results were shown in the analyses of 30-day all-cause mortality (As is shown in Table 4), although the hazard ratios in Q2 and Q3 groups were non-significant, which seemed probably due to inefficient power with the relative small sample size in patients with T2DM.

**Table 4:** Adjusted hazard ratios for heart rate at admission in patients with or without T2DM

	**Admission heart rate(b.p.m.)**						
	**Q1**	**Q2**, **HR(95%CI)**	**P-value**	**Q3,HR(95%CI)**	**P-value**	**Q4,HR(95%CI)**	**P-value**
**Patients without T2DM**							
All-cause mortality	1.00	1.44 (1.13-1.84)	0.003	1.41 (1.10-1.80)	0.007	2.30 (1.85-2.87)	<0.001
Cardiovascular events	1.00	1.39(1.10-1.75)	0.005	1.42(1.22-1.78)	0.003	2.30(1.87-2.83)	<0.001
**Patients with T2DM**							
All-cause mortality	1.00	1.70(0.84-3.41)	0.139	1.84(0.95-3.59)	0.072	2.56(1.38-4.73)	0.003
Cardiovascular events	1.00	1.75(0.92-3.33)	0.086	1.92(1.05-3.48)	0.033	3.00(1.76-5.14)	<0.001

However, of note, there was a significant interaction effect of HR and diabetic state on 30-day CVE mortality (P = 0.035), which indicated that increased heart rate at any level is more deleterious for CVE in diabetic than non-diabetic individuals. The potential interaction effect on all-cause mortality was not found (P = 0.126).

## Discussion

Our study is the first one to assess the association between admission HR and all-mortality and CVE in STEMI patients when accompanied with or without T2DM. The main results are summarized as follows:

1. In STEMI patients accompanied with T2DM, admis-sion HR, 30-day all-cause mortality and rate of CVE was higher compared with those without T2DM.

2. Admission HR was an independent risk factor of prognosis in STEMI patients with or without T2DM.

3. After controlling for baseline and in-hospital therapeutic confounders, the prognostic effect of HR was different between patients with T2DM and without T2DM. In the T2DM patients, the hazard ratios in Q2 and Q3 groups were non- significant, however, it seemed probably due to inefficient power with the relative small sample size. Hazard ratios in each increased HR group were larger in individuals with than without T2DM, and there was a significant interaction effect of HR and diabetic state on 30-day CVE mortality (P = 0.035), which indicated that increased heart rate at any level is more deleterious for prognosis in diabetic than non-diabetic individuals.

Previous studies have shown a positive association between elevated HR and increasing risk of cardiovascular disease (CVD), all-cause and cardiovascular mortality in general people and in patients with various cardiovascular diseases [1,3,14-16], especially AMI. Myocardial ischemia results from an imbalance between coronary blood flow supply and myocardial metabolic demand. HR and other determinants highly affect myocardial oxygen demand [17], meanwhile, HR is the major determinant of the supply of blood flow. Some studies [18] have shown that elevated HR can stimulate the arterial wall, affect local hemodynamic environment [19,20] activate inflammation [21], disturb the imbalance between myocardial demand and supply [22], and disrupt atherosclerotic plaques [23].

Concomitant T2DM then increases the aforementioned influence further when compared with patients without T2DM, propensity to worse prognosis. First, hyperglycemia may play an important role by several pathophysiological mechanisms [24-27]. Secondly, these patients have higher HR [28].

Some epidemiologic studies suggest the relationship between HR and outcome in diabetic patients. Stettler et al. [29] have reported the prognostic role of HR in patients with T2DM. In the Bremen Diabetes study, higher HR was also related to an increase of cardiovascular death [30]. However, Anselmino et al. [31] reported that in patients with stable CAD, the association between resting HR and CVE can be found in those with diabetes, but not in nondiabetic patients. It’s unknown if the difference retains in acute settings, such as STEMI. Our observation performed after separating T2DM from non-T2DM in STEMI patients, which showed that HR was an independent risk factor of prognosis (P < 0.05) regardless of T2DM.

Cardiac autonomic neuropathy (CAN) is a common chronic complication of T2DM [32], and manifests an increase in HR and a reduction in HR variability, that confer higher morbidity and mortality to diabetic patients [33,34]. CAN may contribute to the differences identified in the present study. High HR is not considered to diagnose CAN by itself, but it can reflect a relative imbalance in the sympathetic activity and vagal impairment [32]. It appears that T2DM and autonomic dysfunction are causally related, and a higher HR, elevated high blood pressure, and ischemia burden may be intermediate accelerators [35]. So that, T2DM may strengthen the predictive effect of HR by CAN, but we also need further study of the internal mechanism to confirm this hypothesis.

Our finding in more than 7 000 patients pointed out that HR at admission was an independent predictor of short-term adverse outcome, including cardiovascular and all-cause mortality despite the high level of treatment with beta-blockers. The results emphasized the different of prognostic effect of HR between the two kinds of population, meanwhile. However, more prospective studies are needed to confirm these findings in larger clinical registries.

### Study limitation

First, the study is a subgroup analysis of an existing material, and thereby suffers from limitations and biases of such material. It is not totally equivalent to the real settings. Second, we only observed the all-cause mortality and CVE in 30 days; a longer follow-up may declare more information and elucidate the hypothesis generated in this report. Third, we don’t take blood glucose or glycolated hemoglobin level into account, which reflect the therapeutic effect of T2DM, and may effect the degree of CAN. And the HR in our database is only gained at admission rather than on-treatment or discharge value simultaneously. At last, the sample size of T2DM patients was far less than patients without T2DM (~1/8), and seemed to preclude statistical significance. A larger sample will be needed to improve the statistical power and find out the truth.

## Conclusion

In conclusion, resting HR affects 30-day all-cause mortality and CVE in STEMI patients no matter with or without T2DM. The adverse effect of HR is more obvious in diabetic than non-diabetic individuals. When researching HR and cardiovascular prognosis, T2DM status must be considered. This is also useful in clinical study and treatment.

## Competing interests

The authors declare that they have no competing interest.

## Authors’ contributions

LL and ZJ conceived the origninal idea and designed the research. ZH, LY and TH performed experiments and analyzed results. ZH and YY wrote the manuscript. All authors read and approved the final manuscript.

## Pre-publication history

The pre-publication history for this paper can be accessed here:

http://www.biomedcentral.com/1471-2261/12/104/prepub
